# Rapid evolutionary change of common bean (*Phaseolus vulgaris *L) plastome, and the genomic diversification of legume chloroplasts

**DOI:** 10.1186/1471-2164-8-228

**Published:** 2007-07-10

**Authors:** Xianwu Guo, Santiago Castillo-Ramírez, Víctor González, Patricia Bustos, José Luís Fernández-Vázquez, Rosa Isela Santamaría, Jesús Arellano, Miguel A Cevallos, Guillermo Dávila

**Affiliations:** 1 Programa de Genómica Evolutiva, Centro de Ciencias Genómicas, Universidad Nacional Autónoma de México, Apartado Postal 565-A, C.P 62210, Cuernavaca, Morelos, México; 2 Programa de Genómica Funcional de Eucariotes, Centro de Ciencias Genómicas, Universidad Nacional Autónoma de México, Apartado Postal 565-A, C.P 62210, Cuernavaca, Morelos, México

## Abstract

**Background:**

Fabaceae (legumes) is one of the largest families of flowering plants, and some members are important crops. In contrast to what we know about their great diversity or economic importance, our knowledge at the genomic level of chloroplast genomes (cpDNAs or plastomes) for these crops is limited.

**Results:**

We sequenced the complete genome of the common bean (*Phaseolus vulgari*s cv. Negro Jamapa) chloroplast. The plastome of *P. vulgaris *is a 150,285 bp circular molecule. It has gene content similar to that of other legume plastomes, but contains two pseudogenes, *rpl*33 and *rps*16. A distinct inversion occurred at the junction points of *trn*H-GUG/*rpl*14 and *rps*19/*rps*8, as in adzuki bean [1]. These two pseudogenes and the inversion were confirmed in 10 varieties representing the two domestication centers of the bean. Genomic comparative analysis indicated that inversions generally occur in legume plastomes and the magnitude and localization of insertions/deletions (indels) also vary. The analysis of repeat sequences demonstrated that patterns and sequences of tandem repeats had an important impact on sequence diversification between legume plastomes and tandem repeats did not belong to dispersed repeats. Interestingly, *P. vulgaris *plastome had higher evolutionary rates of change on both genomic and gene levels than *G. max*, which could be the consequence of pressure from both mutation and natural selection.

**Conclusion:**

Legume chloroplast genomes are widely diversified in gene content, gene order, indel structure, abundance and localization of repetitive sequences, intracellular sequence exchange and evolutionary rates. The *P. vulgaris *plastome is a rapidly evolving genome.

## Background

Chloroplasts are derived from an endosymbiotic cyanobacterium that invaded the eukaryotic cell a billion years ago. During the evolutionary process from endosymbiont to contemporary organelles, the cyanobacterium lost the bulk of its genome and retained the genes encoding the photosynthesis machinery and the components of several chemical pathways. During this process, it also acquired many host-derived properties and was thus transformed into a distinct organelle: the chloroplast.

Angiosperm chloroplast genomes present a similar gene content and gene order. They are circular molecules that can also be present in linear forms with multiple copies, ranging in size from 120 kb to 160 kb, but usually around 150 kb with about 90–110 unique genes [2]. A pair of large inverted repeats (IR) about 21–28 kb in length divides the genome into one large single-copy region (LSC) and one small single-copy region (SSC). rRNA genes are always located in IR regions.

Despite the overall conservation of plastomes, genomic diversification was also experienced in many respects. Many genes were lost phylogenetically, independently in parallel or uniquely lost in a particular species [3]. An extreme example is the cpDNA of the parasite plant *Epifagus virginiana*, which lost 13 tRNA genes and retained only 60 genes so that the genome was reduced to 70 kb [4]. It was found that several kinds of inversions interrupted the gene order of the plastome [5-11]. They are generally associated with specific lineages and thus could be a sign of important events in evolutionary diversification [12,13].

Sequence duplication is another feature of some land plant chloroplast genomes. For example, *Pelargonium × hortorum *contains some large duplicated fragments, including several genes, and numerous simple repeats as well as a tremendous extension of IR (75 kb) [14]. Definite evidence supporting transposition within plastid genomes is lacking, but intramolecular recombination mediated by short direct repeats has been reported [15].

The chloroplast genes have been extensively used to study the phylogenetic relationships at several taxonomic levels, especially in the analysis of basal clades, mainly because they have slower mutation rate in comparison with the nuclear genes [16]. The Fabaceae (legume) family is one of the largest and more diverse angiosperm families. It comprises about 20,000 species, which are distributed essentially in tropical regions. Chloroplast-derived markers have been used to study the evolutionary relationship between some legume plants (Fabaceae) [17-21]. However, to date, only the sequences of three legume chloroplast genomes have been reported: *Lotus japonicus*, *Glycine max*, [22,23] and *Medicago truncatula *(AC093544, unpublished). The common bean, *Phaseolus vulgaris*, is a major food crop, domesticated independently in two sites: Mesoamerica and South America[24]. The physical map of its chloroplast genome was published in 1983 [25] and some small pieces of the chloroplast genome were sequenced to study domestication [26] and phylogeny issues. Here we report the chloroplast sequence of *P*. *vulgaris *cv. Negro Jamapa. A comparative analysis of this sequence with other legume chloroplast genomes indicates that these genomes are highly diversified in sequence and organization. Moreover, we provide evidence that one plastome (*P*. *vulgaris*) evolved faster than another (*G. max*) at the genomic and gene levels, which could be the consequence of pressure coming from both mutation and natural selection.

## Results

### General features of the genome

**Figure 1:**
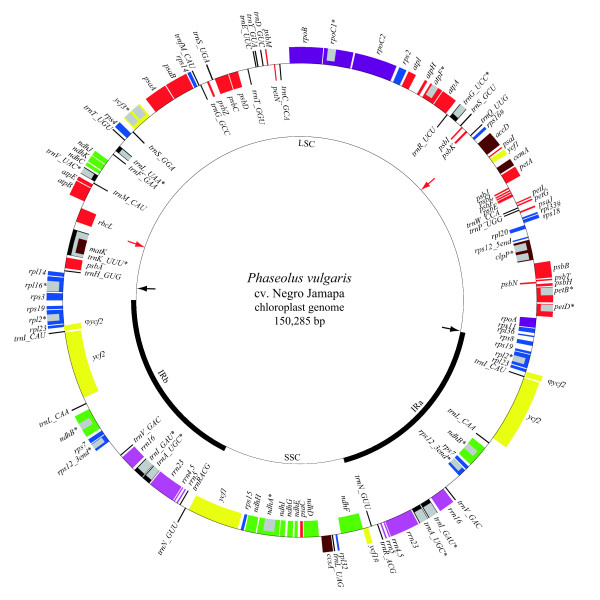
Schematic map of the *Phaseolus vulgaris *plastome. Genes on the outside of the map are transcribed in the clockwise direction and those on the inside are transcribed in the counterclockwise direction. Genes containing introns are indicated by an asterisk. Pseudogenes and incomplete genes are signified by #. Genes are color-coded by function, as shown: blue, ribosomal proteins; red, photosynthesis system; black, transfer RNAs; green, NADH dehydrogenases; yellow, *ycf*; purple, RNA polimerases; light purple, ribosomal RNAs; grey, intron; brown, others. The inner circle shows the quadripartite structure of the plastome. The arrows depict the boundaries of inversions: red arrow indicating the 51 kb-inversion; black arrow indicating the inversion between *trn*H-GUG/*rpl*14 and *rps*19/*rps*8.

The genome of *P. vulgaris *chloroplast is a circular molecule of 150,285 bp that contains an identical IR of 26,426 bp, separated by an LSC of 79,824 bp and an SSC of 17,610 bp (Fig. 1). The noncoding regions, including both introns and intergenic regions, comprises 40.4% of the genome. The overall A+T content for the genome is 64.6% in contrast to 68.7% for the noncoding regions. rRNA genes and tRNA genes have the lowest A+T composition with 45.1% and 47.6%, respectively. A total of 127 genes were assigned to the genome, 108 of which were unique and 19 were duplicated in IR regions. The unique genes included 75 coding-protein genes, 30 tRNA genes, and 4 rRNA genes. There were 17 genes containing one or two introns, six of which were tRNA genes.

### Gene content

The gene content of chloroplast genomes of *P. vulgaris*, *G. max, L. japonicus*, and *M. truncatula*, the legume chloroplast genomes sequenced up to date, was similar. All lacked the *rpl*22 genes and *inf*A, which occurred in other flowering plants. A distinctive characteristic of the *P. vulgaris *chloroplast genome was the presence of two pseudogenes: *rps*16 and *rpl*33. *rps*16 is an intron-containing gene present as a functional gene in both *L. japonicus *and *G. max *but absent in *M. truncatula*. In *P. vulgaris, rps*16 has several features that define it as a pseudogene: firstly, it contains four stop-codons within the second exon; secondly, the gene lacks a functional motif located from the positions 16 to 47 of the amino acid sequence (comparing with the soybean sequence); finally, its initial amino acid is not ATG but ATA. The second pseudogene, *rpl*33, has three stop-codons within its CDS and possesses a GTC as the initial codon. To determine if the stop-codons in these pseudogenes were "corrected" during the RNA-editing process, we compared their sequence against an EST library of *P. vulgaris *cv Negro Jamapa [27]. A cDNA with a perfect match to *rpl*33 sequence was found, indicating that this pseudogene was transcribed and that the stop codons were not edited in its mRNA. In contrast, the *rps*16 sequence was not represented in this library. To demonstrate that the presence of these pseudogenes is not a peculiarity of the bean cultivars that we used in this work, the regions containing *rps*16 or *rpl*33 from 10 other varieties of *P. vulgaris*, belonging to two different domestication centers, were amplified by PCR and the products were sequenced. They gave the same sequence, except for 1–3 SNPs (not shown), indicating that their presence is a common characteristic of the species. *P. vulgaris, G. max*, and *L. japonicus *chloroplast genomes contained 21 unique introns. However, *M. truncatula *lacked intron 1 of *clp*P and the intron present in the 3'-end of *rps*12.

### Gene order

**Figure 2:**
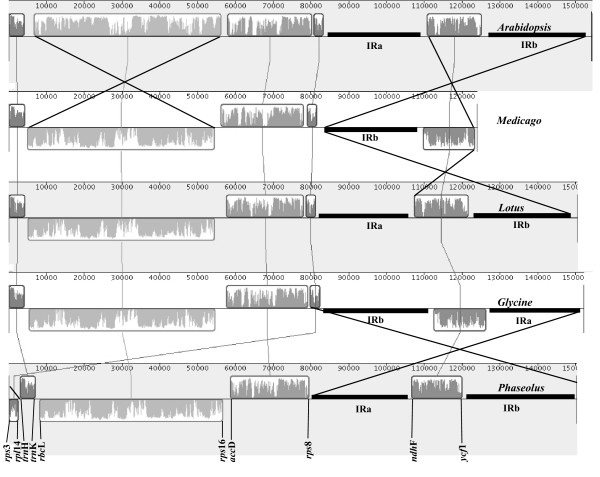
Gene order comparison of the legume plastome, with *Arabidopsis *as a reference, is principally produced by MAUVE. The boxes above the line represent the gene complex sequences in clockwise direction and the boxes below the line represent those sequences in the opposite direction. The gene names at the bottom indicate the genes that are located at the boundaries of the gene complex of the *P. vulgaris *plastome.

Each one of four-sequenced legume cpDNAs possessed its own genome structure (Fig. 2). In comparison with the *Arabidopsis *chloroplast genome (outgroup), *L. japonicus *chloroplast genome has almost the same gene order, except for a 51-kb inversion extending from *rbc*L to *rps*16 in the LSC region, which is present in most taxa of the Papilionoideae subfamily of Leguminosae [8,12,22]. In contrast to the plastome of *L. japonicus*, *G. max *cpDNA seems to have a second inversion embracing the region located between LSC and IRs, but is another isomer product of the flip-flop intramolecular recombination present in platomes [28]. *G. max *and *M. truncatula *shared the same gene order but the conspicuous difference between them was the absence of the IRb region in the latter. The *P. vulgaris *cpDNAcontained an inversion at the junction between *trn*H-GUG/*rpl*14 and *rps*19/*rps*8 which was absent in the three other legume chloroplast genomes. We confirmed the presence of this peculiar structure in 10 other *P. vulgaris *varieties originating from Mesoamerican and South American domestication centers, using a concatenated long PCR analysis. This genome inversion has also been reported in the adzuki bean (*Vigna angularis*) [1] and mung bean (*Vigna radiata*) [8]. These results indicate that the structure found in *L. japonicus *cpDNA was closer to the legume ancestral gene order.

### IR region

The IR in *P. vulgaris *contained 19 complete genes and spanned 26,426 bp, longer than *G. max *(25,574 bp) and *L. japonicus *(25,156 bp). The *P. vulgaris *duplicated region included the whole *rps*19 gene and 572 bp of its downstream sequence, whereas in both *G. max *and *L. japonicus*, the IRs included only a partial fragment of the *rps*19 gene. Thus, the length increase of IR was principally attributed to the expansion of the IR region at the junction between IR/LSC.

The junction points of IR/LSC were located in 24 bp from the start base of *rps*3 CDS at one end and 53 bp from the start base of *rps*8 CDS at the other. This was exactly like the adzuki bean[1], indicating that this IR predated the speciation of these two bean species, but after the separation from soybean. The boundaries between SSC/IR are located within the *ycf*1 gene and for this reason, 505 bp of this gene's 5'-end is repeated. A similar repetition was found in *G. max *(478 bp) and *L. japonicus *(514 bp), which are shorter than the repeat in *Arabidopsis *(1027 bp).

### Indel structure

**Figure 3:**
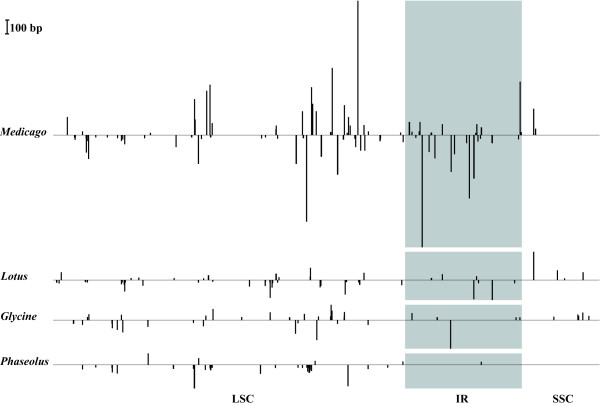
Indel profiles of legume plastomes. Indels were identified by the sequence alignments with Clustal-X [66]. The black bars above the horizontal axis indicate insertion and those below the axis show deletion. The height of the boxes represents the size of indel fragments. The sequence order is shown as in *P. vulgaris*. The shadow region represents one IR and another IR was removed from the figure.

A number of insertions/deletions (indels) present on cpDNA homologous regions shared by *M. truncatula*, *G. max*, *L. japonicus*, and *P. vulgaris *were detected by DNA alignments. In Figure 3, indels greater than 20 bp are shown. Indels in *P. vulgaris *were principally concentrated at the LSC region, only one was in IRs (24 bp); but deletion was more common than insertion in its cpDNA, which resulted in the reduction of the genome size. In contrast, *M. truncatula *had more and larger indels than other legume plants, and even lost one copy of IR. A large part of the indels was located at the intergenic regions or introns but some of them lay within genes, common in *ycf*1, *ycf*2, *psa*A, *rps*16, *rps*18, and *acc*D.

### DNA repeat analysis

All repeated sequences of 20 bp or larger with 100% identity were examined in each of the four legume chloroplast genomes. *M. truncatula *had the largest number of repeats, as described by Saski [23], whereas *P. vulgaris *had the least. Repeats were generally located within the intergenic regions or within introns; however, some of them were present in genes, usually *ycf*1, *ycf*2, *psa*A, and *acc*D.

The biggest direct repeat found in *P. vulgaris *cpDNA was a 287-bp duplication of an internal fragment of *ycf*2 (ψ*ycf*2, Fig. 1). In *P. vulgaris *and *G. max*, this repeat had the same size, while in *L. japonicus *this segment was a little smaller, 265 bp. These two copies in *P. vulgaris *were identical, as well as in *G. max *and in *L. japonicus*, but in *M. truncatula*, it already diverged, sharing 56% of identity. Palindromic repeats were normally situated within intergenic regions and in proximity to the gene end. In *P. vulgaris*, an identical 20-bp-sized palindromic sequence was found within 70 bp from the ends of genes *trn*H-GUG, *ycf*3, and *ycf*1, indicating that they could have the same function.

### Tandem-repeat analysis

The distribution of tandem repeats in the legumes cpDNAs is shown in Table 1. *Phaseolus *has five groups of tandem repeats, the smallest number of the sequenced legume cpDNAs. One repetitive unit of 16 bp was duplicated four times within the IR region and was located close to the boundaries of IR/LSC (coordinate positions: 80116–80179 and 149929 – 149992). The alignment of this region with the corresponding sequences of other legume cpDNAs available from Genbank showed that adzuki bean possessed this duplicated tandem repeat, but with three repeated units each. *G. max *and *L. japonicus *lost this sequence. However, *M. truncatula *had only one 16-bp unit with 75% identity at this position.

**Table 1:** Distribution of tandem repeats (> 15 bp with 80% identity between copies) in four legume plastomes

	Initial position	Final position	Size (≥ 15 bp)	Copies	Identity (≥ 80%)	Position related genes
*Phaseolus*	66513	66572	15	4	80	*psa*J/*rpl*33
	65733	65783	17	3	92.2	*trn*W/*trn*P
	80116	80179	16	4	98	*rps*8/*rps*19, or *rps*3/*rps*19
	85700	85762	21	3	88.9	*ycf*2
	88119	88172	18	3	88.9	*ycf*2
*Lotus*	1694	1765	24	3	81.9	*psb*A/*trn*K
	14487	14543	19	3	84.2	*trn*L/*trn*T
	17838	17888	17	3	86.3	*ycf*3, intron
	24441	24492	26	2	100	*trn*G/*ycf*9
	47831	47878	16	3	96	*atp*H/*atp*F
	54191	54265	25	3	80	*psb*K/*trn*Q
	87031	87093	21	3	91	*ycf*2
	89444	89524	27	3	95	*ycf*2
	106513	106572	20	3	83.3	*trn*N/*ycf*1
	109580	109642	21	3	84.1	*ndh*F/*rpl*32
*Glycine*	28572	28640	23	3	81.2	*psb*D/*trn*T
	51493	51555	21	3	84.1	*atp*A/*trn*R
	51753	51818	22	3	86.4	*trn*R/*trn*G
	58325	58396	24	3	80.6	*acc*D/*psa*I
	64627	64674	24	2	96	*pet*G/*trn*W
	66304	66345	21	2	100	*rpl*33/*rps*18
	68386	58429	22	2	100	*clp*P/*rps*12_5'-end
	81892	81954	21	3	85.7	*rpl*16,*rps*3
	82665	82718	18	3	85.2	*rps*3,*rps*19
	83848	83901	18	3	85.2	*rpl*2, intron
	88334	88396	21	3	91	*ycf*2
	89622	89663	21	2	100	*ycf*2
	90774	90827	18	3	85.2	*ycf*2
	108203	108252	25	2	96	*trn*N/*ycf*1
	123651	123710	20	3	85	*trn*L/*rpl*32
	127141	127190	25	2	96	*ycf*1
*Medicago*	13248	13319	24	3	84.7	*rps*15/*ycf*1
	17087	17158	24	3	100	*ycf*1
	18922	19013	46	2	100	*ycf*1/*trn*N
	18847	19031	37	5	84.3	*ycf*1/*trn*N
	19100	19219	60	2	100	*ycf*1/*trn*N
	27448	27617	85	2	93	*rrn*16/*trn*V
	36490	36669	60	3	98	*ycf*2
	37267	37401	45	3	83.7	*ycf*2
	38869	38940	36	2	100	*ycf*2/*trn*I
	38954	38997	22	2	100	*ycf*2/*trn*I
	39247	39368	61	2	89	*trn*I/*rpl*23
	55590	55875	143	2	100	*clp*P/*rps*12_5'-end
	55807	55920	57	2	88.6	*clp*P/*rps*12_5'-end
	56146	56265	24	5	95	*clp*P/*rps*12_5'-end
	56392	56466	25	3	100	*clp*P/*rps*12_5'-end
	58382	58441	15	4	90	*rps*18
	58799	58867	23	3	81.2	*rps*18/*rpl*33
	65523	65586	32	2	96.9	*cem*A/*psa*I
	67538	67702	33	5	91.5	*acc*D
	67639	67818	60	3	98	*acc*D
	68026	68214	63	3	100	*acc*D
	68251	68322	24	3	88.1	*acc*D
	68311	68436	63	2	93	*acc*D
	68577	68624	24	2	86.7	*acc*D
	68907	68954	24	2	96	*acc*D
	69341	69422	41	2	96	*acc*D/*trn*Q
	91311	91394	28	3	81	*trn*C/*pet*N
	99689	99742	18	3	92.6	*psb*Z/*trn*G
	105175	105222	24	2	98	*psa*A

*M. truncatula *had a similar number of reverse and palindrome repeats to other legume plastomes but had a higher proportion of tandem repeats (2% of its genome), compared to other legume cpDNAs. The majority of tandem repeats were located within coding regions of *acc*D, *ycf*1, and *ycf*2 genes and into intergenic regions between *clp*P/*rps*12-5'end and *ycf*1/*trn*N. For example, the *acc*D gene contained seven kinds of repeats in tandem from two to five copies. Of all tandem repeats found in *M. truncatula*, only one (coordinate number: 37267–37401) in *ycf*2 was, to a different extent, shared by all the legume plastomes. Consensus sequences of repetitive units of each tandem repeat present in *M. truncatula *cpDNA were obtained and searched in the other legume cpDNAs. The consensus sequences of repeats within *ycf*1, *ycf*2, *rps*18, and *psa*A were found in the other genomes but as single sequences (not repeated).

**Figure 4:**
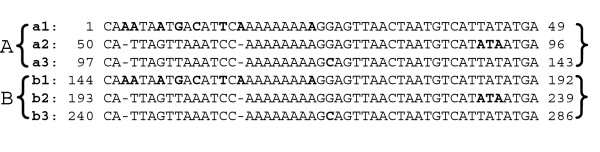
Largest tandem repeats in *Medicago *at the coordinate of 55590 and 55875. Repeats A and B are respectively composed of smaller tandem repeats, a1-3 and b1-3.

The largest tandem repeat in *M. truncatula*, spanning 286 bp, was situated at the end of *clp*P (coordinates 55590 and 55875), and it was exclusively found in cpDNA of this plant. It consisted of two identical tandem copies of 143 bp, repeats A and B (Fig. 4). In fact, this segment was also composed of six copies of a smaller repeated unit of approximate 48 bp, of which some copies were altered by a few bases (a1, b1) or had some base insertions (a2, a3, b2, b3), but the backbone was conserved. This structure suggests that the 48 bp was first duplicated two consecutive times, and then each of these units underwent some degree of diversification to form the 143 bp. More recently, this last element was duplicated. Similar situations were found in the *acc*D gene and the intergenic region *ycf*1/*trn*N.

### Phylogenetic analysis

**Figure 5:**
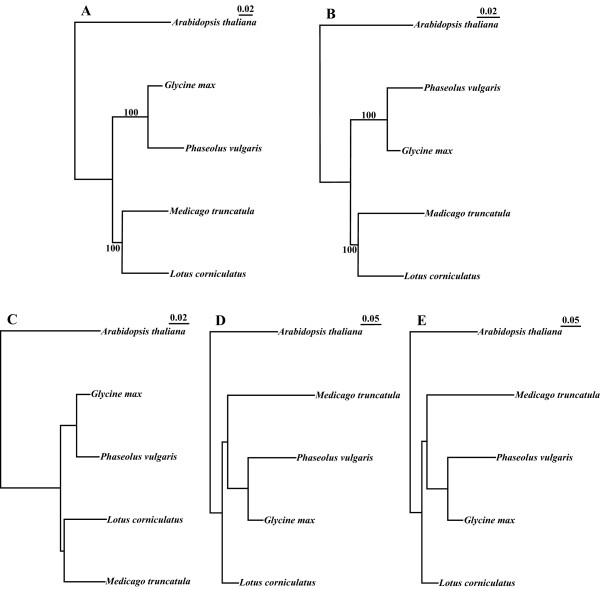
Diagrams of phylogenetic trees. Topology A was deduced from all the genome sequences and B was based on all the genes. C, D, E are different topologies of individual gene phylogenies.

Legume chloroplast phylogenies were established using a phylogenomic approach and the phylogenetic information of individual genes. In our analyses, we always used the *A. thaliana *chloroplast genome as the outgroup. From the phylogenomic perspective, we made two large alignments: one with all homologous regions of the five cpDNAs but excluding the paralogous regions, and the other, by pasting together the individual alignments of 102 individual genes. Both gave similar tree topologies, forming two subgroups with a bootstrap value of 100: *Phaseolus *with *Glycine *and *Medicago *with *Lotus *(Fig. 5a, b), which correspond to the previously well-established phylogeny [21]. It was apparent that, in the group of *Phaseolus *with *Glycine, Phaseolus *has accumulated more substitutions than *Glycine*, thus *Phaseolus *diversified much faster (2.3 times), while *M. truncatula* and *L. japonicus* has a similar substitution rate (Fig. 5a, b). To support the phylogeny obtained with genomes, we also did phylogenies with each of the 75 protein-encoding genes (*rps*12 is a divided gene: its -5' and 3'ends were considered here as two genes because they are encoded at different loci; *ycf*4 was not used due to the absence in *M. truncatula* and *L. japonicus* plastomes). Ribosomal RNA and transfer RNA genes were not included because of fewer base substitutions. 60 protein-coding genes produced phylogenies with bootstrap values higher than 50. These 60 phylogenies were classified into five topologies: three of them were obtained more frequently (Fig. 5c–e) and the other two topologies were only supported by single genes (not shown). The most frequent topology, representing 28 genes (47%), matched the topology obtained with phylogenomic analysis. Topologies D and E represent phylogenies of 18 (30%) and 12 (20%) genes, respectively. In all of these topologies *G. max *and *P. vulgaris *made a cluster, but *M. truncatula *or *L. japonicus *differed in the relation to *A. thaliana*, the outgroup. It is important to point out that phylogenies obtained with *mat*K and *rbc*L (topology D), two genes commonly used in plant phylogenic analysis, do not fit the genome-based topology, suggesting that care must be taken in interpreting data obtained with these gene-markers.

### Relative evolutionary rate

The genome-based phylogenies indicate that legume chloroplast genomes change at different rates. To identify which genes and to what extent these genes contribute to the overall evolutionary rate, a relative rate test was performed. The relative rates between *Phaseolus *and *Glycine *and those between *Medicago *and *Lotus *in K, Ks, and Ka of all protein-coding genes were determined. Considering that the outgroup plastome could affect, to some extent, the analysis, each relative test employed one of three different genomes alternatively as an outgroup. The relative rate tests between *P. vulgaris *and *G. max *were evaluated using as a reference species, *A. thaliana, M. truncatula*, or *L. japonicus*. Similarly, the relative rate tests between *M. truncatula *and *L. japonicus *were calculated using *A. thaliana, P. vulgaris*, or *G. max *as reference group.

**Figure 6:**
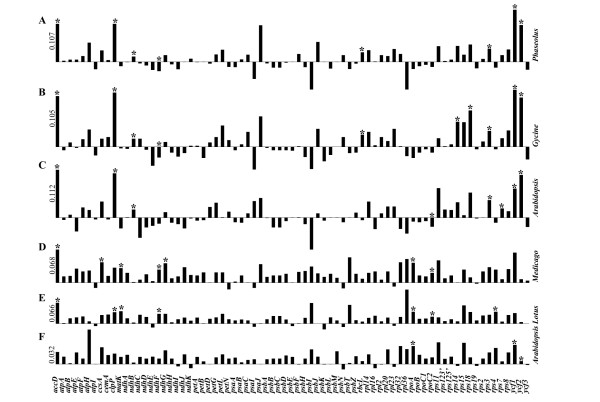
Diagrams of differences in evolutionary rates of "K", the number of nucleotide substitutions per site, of 75 protein-coding genes. Panels A, B, and C represent the variances in relative rates between *Medicago *and *Lotus *using the reference plastomes, respectively, as *Phaseolus, Glycine*, and *Arabidopsis*. Panels D, E, and F show those between *Phaseolus *and *Glycine *using the reference plastomes, respectively, as *Medicago*, *Lotus*, and *Arabidopsis*. The height of the black bar denotes the value of variances (the first bar showed the value, as a scale of this panel). The bars above the axis mean *Medicago *with higher substitution rates than *Lotus *in Panels A, B, and C or *Phaseolus *with higher substitution rates than *Glycine *in Panels D, E, and F and the bars below the axis represent the opposite case. The asterisk is a sign of significant difference (P < 0.05).

In the comparing *P. vulgaris *and *G. max*, we found a number of *P. vulgaris *genes with a strong tendency to evolve faster, despite the different reference species used (Fig. 6). All the genes with statistical significance (p < 0.05) K, Ka, and Ks values also produced the same results (Fig. 6, Tables 2 and 3). We therefore concluded that there was faster diversification of the *P. vulgaris *plastome than *G. max *at the genomic level. Comparing *M. truncatula*-*L. japonicus*,12 genes evolved at a significantly different rate (K), 10 of which accumulated more substitutions in *M. truncatula *(Fig. 6A, B, and 6C), and two of which had more substitutions in *L. japonicus*.

**Table 2:** Synonymous (Ks) and Nonsynonymous (Ka) substitution rates of *P. vulgaris *and *G. max*.

	*Arabidopsis *as a reference		*Lotus *as a reference		*Medicago *as a reference	
	
	Ka	Ks	Ka	Ks	Ka	Ks
	Pha.^#^/Gly.	Pha./Gly.	Pha./Gly.	Pha./Gly.	Pha./Gly.	Pha./Gly.
*acc*D	---	---	0.1769/0.1234	0.1265/0.0572	0.2587/0.2096	0.2354/0.1512
*ccs*A	---	---	---	---	0.1061/0.0782	---
*clp*P	---	---	0.0603/0.0284	---	---	---
*mat*K	---	---	0.1692/0.1371	---	0.1633/0.1365	---
*ndh*F	---	---	0.1065/0.082	---	0.094/0.0723	---
*ndh*G	---	---	0.0609/0.0323	---	0.0659/0.0309	---
*psb*D	---	---	---	---	---	0.2203/0.1492
*rpo*A	0.1356/0.1026	---	0.0803/0.0552	---	0.0747/0.0493	---
*rpo*B	0.0685/0.0562	---	0.0535/0.0425	---	0.0472/0.0361	---
*rpo*C1	---	---	0.0497/0.0375	---	---	---
*rpo*C2	---	---	0.1123/0.0961	---	0.1089/0.093	---
*rps*15	0.1906/0.1207	---	0.1166/0.0613	---	---	---
*rps*2	---	---	0.0669/0.0442	---	---	---
*rps*4	---	---	0.0635/0.0389	---	---	---

^# ^Pha. and Gly. represent respectively *Phaseolus *and *Glycine*.

**Table 3:** Synonymous (Ks) and Nonsynonymous (Ka) substitution rates of *M. truncatula *and *L. japonicus*.

	*Arabidopsis *as a refernce		*Glycine *as a reference		*Phaseolus *as a reference	
	
	Ka	Ks	Ka	Ks	Ka	Ks
	Med.^#^/Lot.	Med./Lot.	Med./Lot.	Med./Lot.	Med./Lot.	Med./Lot.
*acc*D	0.2822/0.1869	0.2074/0.1222	0.2096/0.1234	0.1512/0.0572	0.2587/0.1769	0.2354/0.1265
*atp*A	---	---	0.0184/0.0092	0.2455/0.3494*	0.0226/0.0134	---
*atp*B	---	---	0.0232/0.0128	---	---	---
*atp*H	---	---	---	---	0.0218/0	---
*clp*P	0.1803/0.0668	---	0.1503/0.0284	---	0.1734/0.0603	---
*ndh*B	---	0.1297/0.0754	---	0.1189/0.0633	---	0.1126/0.0652
*ndh*E	---	---	---	0.186/0.4588*	---	---
*ndh*F	---	---	---	0.4389/0.5855*	---	0.4925/0.6659*
*pet*B	---	---	---	0.2429/0.3904*	---	---
*psa*B	---	0.3872/0.4844*	---	---	---	---
*rbc*L	---	---	---	---	---	0.5606/0.3989
*rpo*C2	---	0.3959/0.4735*	---	---	---	---
*rps*11	---	---	0.0596/0.0274	---	---	---
*rps*14	---	---	0.0706/0.0219	---	0.0704/0.0308	---
*rps*18	0.1263/0.0718	---	0.1107/0.0397	---	0.1152/0.0648	---
*rps*3	0.1048/0.069	---	0.0862/0.0442	---	0.1013/0.0602	---
*rps*7	0.0392/0.0055	---	0.0332/0.0055	---	0.0417/0.0137	---
*ycf*1	---	---	0.178/0.0946	0.2648/0.0946	0.2192/0.1205	0.3529/0.1409
*ycf*2	0.161/0.0674	0.1576/0.0681	0.1487/0.054	0.1481/0.0535	0.1556/0.0588	0.1511/0.058

* The star signal represents *Lotus *genes with higher substitution rates than *Medicago *genes.

^#^Med. and Lot. indicate respectively *Medicago *and *Lotus*.

In both groups, *P. vulgaris-G. max *and *M. truncatula*-*L. japonicus*, all the *pet*, *psa*, *psb*, and *atp* genes showed no significant difference in substitution rates, and six genes (*acc*D,* ycf*1,* ycf*2,* clp*P*, ndh*F, and* rpo*C2) evolved at different rates (Tables 2 and 3, Fig. 6). Some genes containing significant differences in the group *P. vulgaris-G. max *did not demonstrate significant differences in *M. truncatula*-*L. japonicus*. This result suggests that, in legume plastomes, some genes showed similar evolutionary tendency and others diversified faster in a particular plastome. *acc*D and *ycf*2 presented different rates of both synonymous and nonsynonymous changes, implying that these genes have low functional compromise. Moreover, *acc*D and *ycf*2 had a ω index (Ka/Ks) higher than 1, indicating that they are subjected to a strong diversifying process. The rest of the genes with significant change rates had a w index lower than 1, showing that these genes are under purifying selection.

## Discussion

### Gene order and gene content of legume plastomes

In contrast to the genome organization in *A. thaliana*, most taxa of the subfamily Papilionoideae, including the four species of which plastomes are sequenced, present a 51-kb inversion within the LSC region [12]. Another inversion at the junction points of *trn*H-GUG/*rpl*14 and *rps*19/*rps*8 was only reported to occur in two genera, *Phaseolus *and *Vigna*[1,19,29], indicating that this chloroplast genome arrangement is characteristic of the *Phaseolus*-*Vigna *species complex. The chloroplast genome of *M. truncatula *lacks one IR, a feature shared with other legume tribes such as Carmichaelieae, Cicereae, Galegeae, Hedysareae, Trifolieae, and Vicieae and some genera of other groups [13]. Now, all these tribes form a new clade, IRLC (inverted-repeat-loss clade) [30]. Thus, the four-sequenced plastomes represent three types of plastome structure, suggesting that the cpDNA organization is very diverse in legume plants.

Legume cpDNAs do not contain *rpl*22 [31,32] and *inf*A [33] genes, indicating that they were phylogenetically lost from this lineage. A specific character of *P. vulgaris *cpDNA is the presence of the two pseudogenes *rps*16 and *rpl*33. The first is functional in *L. japonicus *and *G. max *but is lost in *M. truncatula *[23,32]. The cpDNAs of other land plants, *Selaginella uncinata*, *Psilotum nudum*, *Physcomitrella patens*, *E. virginiana*, and *Eucalyptus globules*, lost this gene independently [4,34,35]. *rpl*33 is a functional gene basically present in all land plant chloroplasts, except in *S. uncinata*. These data suggested that *P. vulgaris *cpDNA is still undergoing genome reduction.

The *acc*D gene encodes an acetyl-coenzyme A carboxylase subunit similar to prokaryotic *acc*D in structure[36], and is the most variable gene present in legume chloroplasts. Its size is widely different: 1299 bp in *G. max*; 1422 bp in *P. vulgaris*; 1506 bp in *L. japonicus*, and 2142 bp in *M. truncatula*. *Medicago *has the largest *acc*D of prokaryotic form, containing seven kinds of tandem repeats and one 43-bp-sized separate direct repeat situated between two conserved regions. We did a BLAST-search with the *acc*D gene against the EST bank of *M. truncatula*. One tentative consensus segment of 9334 bp (TC106672) was found to contain the identical sequences of chloroplast genes *trn*S-GCU, *trn*Q-UUG, *psb*I, *psb*K, *acc*D, *psa*I, *cem*A, and *pet*A, indicating that these genes are transcribed. Nevertheless, the large amount of tandem repeats present in the *M. truncatula acc*D gene calls into question its functionality.

Another landmark of the legume plastomes is the duplication of a portion of *ycf*2. The duplicated segment, named ψ*ycf*2, was first identified as a pseudogene in *Vigna angularis *[1]. It is present in the same relative position in the legume plastomes analyzed here. In *G. max*, *P. vulgaris *and *L. japonicus*, ψ*ycf*2 is identical to its copy within *ycf*2, but in *M. truncatula *they are very divergent (60 % of identity). This result indicates that the last common ancestor of these plants already had this duplication and gene conversion occurred in the plastomes containing IR.

### Nature of tandem repeats

The sequence and distribution of repetitive elements are characteristic of each chloroplast genome, and they can be classified in two broad categories: large repeats and short dispersed repeats (SDRs). Both categories can be found in different proportions in chloroplast genomes. *Oenothera *and *Triticum *chloroplasts contain some dispersed repeats, but 20% of the *Chlamydomonas reinhardtii *plastome consists of repeated sequences, many of them are tandem repeats (TR) [37-39]. In legume plastomes, clear differences reside in the number, location, and sequence of TR. *M. truncatula *possess a plastome with greater number and larger TRs, and *P. vulgaris *has a plastome with fewer TRs.

Usually, TRs are classified as a subcategory of SDRs, but our analysis of the legume chloroplast genomes shows that TRs have a different origin from the rest of the SDRs. The repetitive unit of an SDR family is dispersed throughout the genome and different members of an SDR family share high identity. In contrast, the repetitive unit of a TR is not dispersed, and the consensus sequence of each TR has low identity with the consensus sequences of other TRs, with the exception of some repeats with low complexity (*i. e*. ATATAT). In other words, each TR is specific to a site.

Multi-alignments among plastomes frequently show that a repetitive consensus unit of a TR can be found in other chloroplast genomes at similar positions without duplication, or the region containing corresponding sequences are completely deleted from a specific plastome. Moreover, some small insertions from 7 bp to 21 bp are the duplication events of one of the flanking sequences in a specific plastome to form a small TR (only two tandem units). On the other hand, more complicated TRs by consecutive duplication, as shown in Figure 4, also exist in other sites of the plastome. Taking together our observations, we conclude that TRs came from *in situ *sequences and do not share the same origin of dispersed repeats.

We propose that homology-facilitated illegitimate recombination is the mechanism that creates TRs. The reasons are: 1). TRs arise from *in situ *sequences, actually from 7 bp to 143 bp long in the present study; 2) About 4–17 bp initial bases of some larger insertions are the iteration of their flanking sequence; 3) There are many copies of the plastome in a cell, both in circle and in linear forms, which provide the opportunity of such recombination; 4) Homology-facilitated illegitimate recombination is corroborated by the gene transformation in the chloroplast of *Acinetobacter *sp. [40]. Recombination mediated by short direct repeats was reported in wheat chloroplast [15].

### Intracellular sequence exchange

Recently, Kami reported the sequence from a nuclear BAC clone, 71F18, containing a chloroplast-derived DNA of *P. vulgaris *[41]. The sequence comparison between the *P. vulgaris *plastome and the BAC clone showed that two separate regions (*trn*G-*rps*14 in 914 bp, *trn*I-*ndh*B in 7901 bp) in the plastome were linked together in the nuclear genome, with the same similarity (99.01%) to their nuclear homologues. We noted that the nuclear homologues did not contain the insertion in comparison with its plastome sequence, but had 8 deletion segments ranging in size from 8 bp to 583 bp. We therefore postulate that the original fragment transferred from the plastome, likely spanned the whole fragment from *trn*I-GAU to *rps*14 (73 kb), and then some deletions occurred, including the deletion of 64 kb fragment from *trn*L to *psb*Z.

A BLAST-search of the *M. truncatula *plastome sequences with available nuclear genome sequences of this species found that 51% of the plastome is present in the nuclear genome with more than 99% identity. These identified chloroplast-derived segments of the *M. truncatula *nuclear genome can be as large as 25 Kb. One must take into account that we only had the opportunity to explore a partial nuclear genome that is available up to date in Genbank, suggesting that the whole plastome could be found in the nuclear genome if the complete nuclear genome becomes available. If so, it is similar to the case of the rice genome [42], but different from *A. thaliana*, in which the chloroplast-derived fragments found in the nuclear genome have a lesser degree of identity (commonly 92–98%) and the transferred fragments are smaller in size, generally less than 4 kb, indicating that cpDNA transfer occurs earlier in the *A. thaliana *genome. In the rice genome, cpDNAs are continuously transferred to the nuclear genome, which incessantly eliminates them, until an equilibrium is reached [42]. On the other hand, we did not find significant similarity between the plastome of *L. japonicus *and its nuclear genome. There are several hypotheses to explain the gene transfer from chloroplast to nuclear genomes [43]. The most common mechanism of transfer depends on chloroplast lysis, but it is still difficult to elucidate why the nuclear genome of *A. thaliana *did not integrate cpDNA with the same patterns as *M. truncatula *or *O. sativa*.

### Rate of evolutionary change in legume plastomes

There are only a few reports that describe the evolutionary rate of the chloroplast genome [44-46]. In the present study, we demonstrate that one plastome (*P. vulgaris*) globally evolved faster than another plastome (*G. max*), which has not been observed before.

In regard to the evolutionary rate of legume plants, Lavin reported that *Phaseolus *and closely related genera have the fastest substitution rates at the *mat*K locus, within Leguminosae [21]. Delgado-Salinas recently suggested this accelerated substitution rate in *mat*K (within the intron of *trn*K) is related to the formation of the modern Trans-Mexico volcanic belt [47]. We present further evidence here that the *Phaseolus *plastome genomically diversified rapidly. Considering that all the genes in this genome were affected, we deduced that some factor likely impacted this plastome globally, leading to a higher rate of evolutionary change.

Evolutionary rate can be mainly affected by the following factors: generation time, population size, specific mutation rate, and natural selection [48]. The first three factors should influence all the genes of a genome as a whole, whereas the third is able to impinge on specific genes. Generation time is usually considered as an important cause for acting on the evolutionary rate, and has been applied in the elucidation of the discrepancy of evolutionary rates between rodents and other mammals [49], between the plastomes of *Phalaenopsis aphrodite *and grass crops [50], and between rice and maize [46]. However, it cannot be applied to explain the phenomenon in the present study because both *G. max *and *P. vulgaris *are annual crop plants, sharing the same generation time. Population sizes of *G. max *and *P. vulgaris *cultivars seem to be similar because they are important domesticated plants with a highly limited genetic diversity [51]. The divergent mutation rate could be one of the causes of the variance in the substitution rate between *Phaseolus* and *Glycine*. The reasons are: 1) overall Ks in *Phaseolus* is much higher than *Glycine* (see Additional File 1); 2) the sites of synonymous substitution are far from saturation in this plastome (< < 1); 3) and these two crop plants have the same generation time and similar reproductive mode (self-fertilization), which prevents genetic recombination from other plants; and 4) the chloroplast is rarely imported from other compartments of a cell as genetic elements. On the other hand, natural selection should be a factor for the relative rate of specific genes. The present research shows that almost all genes are under a purifying selection (ω < 1). Therefore, we conclude that the different evolutionary rate between *Phaseolus* and *Glycine* is a consequence of the pressures of both mutation and natural selection.

The *M. truncatula *and *L. japonicus *plastomes evolved at a similar rate (K). However, the genes with significant differences showed a remarkably distinct rate: 10 *M. truncatula *genes evolved significantly faster than did their *L. japonicus *counterparts, but two genes, *rpo*C2 and *ndh*F, changed faster in *L. japonicus*. In this case, it seems that the particular reason that leads to faster evolution of some genes in one plastome must be natural selection.

## Conclusion

Plastomes of leguminous plants have evolved specific genomic structures. They have undergone diversification in gene content, gene order, indel structure, abundance and localization of repetitive sequences, intracellular sequence exchange and evolutionary rates. In particular, the *P. vulgaris *plastome globally has evolved faster than that of *Glycine*.

## Methods

### Biological materials

The *P. vulgaris *cultivars used in this work were Negro Jamapa, Pinto V1-114, Kentucky wonder, Carioca, Olathe, Othello, MSU Fleet Wood, Jalo EEP558, and BAT93, derived from the mesoamerican domestication center and Cardinal and Red Kloud, derived from the Andean domestication center.

### Chloroplast DNA extraction, DNA sequencing, and genome annotation

*P. vulgaris *cv. Negro Jamapa cpDNA was isolated from intact chloroplasts using the method reported by Jansen [52]. To construct the shotgun library, DNA was fragmented by nebulization. Fragments between 2 and 5 kb were recovered from 1% agarose gel, blunt-ended, and cloned in pZERO™-2 in its *Eco*RV site (Invitrogen). Recombinant clones were sequenced using the Dye-terminator cycle sequencing kit (Perkin Elmer Applied Biosystems, USA). Sequencing reactions were run in an ABI 3730 sequencer (Applied Biosystems). To seal small gaps, specific regions were amplified by polymerase chain reaction (PCR), and the obtained products were sequenced. Assemblages were obtained using the PHRED-PHRAP-CONSED software [53,54] with a final quality of < 1 error per 100,000 bases. Genome annotation was performed with the aid of the DOGMA program [55]. The start and stop codons and the boundaries between introns and exons for each protein-coding gene were determined by comparison with other published chloroplast genomes using BLASTX [56]. We also annotated the *M. truncatula *plastome because its annotation is not available from Genbank.

### PCR amplification

Concatenated long PCR was adopted to confirm the gene order of the *P. vulgaris *chloroplast genome and to analyze the gene order of closely related bean varieties. Primers for amplifying the whole genome as overlapping segments are shown in Additional File 2. The pairs of primers for the amplification of pseudogenes, *rps*16 and *rpl*33, were: *rps*16F (5'-tgtagcgaatgaatcaatgc-3'), *rps*16R (5'-tgccttactcaatgtttgttc-3'); *rpl*33F (5'-aaattcggagtgaaactcg-3'), *rpl*33R (5'-tctcagtcgactcgctttt-3'). PCR assays were performed in a 25 μl reaction volume containing 250 ng template DNA, 1× reaction XL buffer II, 1.1 mM Mg(OAc)_2_, 200 μM dNTPs, 5 pmol of each primer, and 1 unit of rTth DNA polymerase XL (Perkin Elmer). PCR amplifications were carried out in a 9700 thermocycler (Perkin Elmer) with the following conditions: an initial denaturation at 94°C for 1 min; 30 cycles of denaturation at 94°C for15 s, annealing and extension at 62°C for 3–15 min (depending on the fragment size needed to amplify); and a final extension at 72°C for 7 min.

### Genome analysis

Gene order comparison between the chloroplast genomes of *P. vulgaris *(DQ886273), *A. thaliana *(AP000423),*G. max *(DQ317523),*L. japonicus *(AP002983), and *M. truncatula *(AC093544) was performed with MAUVE [57]. REPuter [58] was used to identify the number and location of direct, reverse, and palindromic repeats of genomes with minimum identical repeat size of 20 bp. Meanwhile, Equicktandem and Etandem [59] were applied to find the distribution of tandem repeats.

### Evolutionary analysis

Genes were defined as homologs with the criterion of E value, 1×10^-12^, in a BLAST search, using as queries the *P. vulgaris *genes against other chloroplast genomes mentioned above [56]. Two big alignments were made. The first one was a multigenome alignment produced by MAUVE [57]. The second one was constructed by two steps: creating the homologous alignments of each of 74 individual protein-encoding genes that had at least one copy in each genome by MUSCLE [60] and then pasting all the individual gene alignments together to form a big one (concatenated alignment). Alignments were edited to exclude gap-containing columns.

A DNA substitution model was selected using Akaike information criterion with Modeltest, version 3.7 [61]. For the alignments described earlier, the General Time Reversible (GTR) model, including rate variation among sites (+G) and invariable sites (+I), was chosen as the best fit. One thousand replicates were generated with SEQBOOT. Phylogenies were constructed using PHYML [62] and DNAPARS and the consensus phylogenetic tree was obtained with CONSENSE. For each of the 74 individual gene alignments, a phylogeny was produced with PHYML, using a nonparametric bootstrap analysis of 100 replicates. TREEDIST was used to estimate how many different topologies there are, but only the topologies with nonparametric bootstrap values higher than 50 were considered. SEQBOOT, DNAPARS, CONSENSE, and TREEDIST were downloaded from the PHYLIP package version 3.61 [63].

The number of nucleotide substitutions per site "K" was calculated with MEGA3 [64]. The number of nucleotide substitutions per synonymous site "Ks" and the number of nucleotide substitutions per nonsynonymous site "Ka" were deduced with yn00 from PAML13.14 [65]. Based on these data, K, Ks, and Ka, a triplet relative rate test was employed to evaluate the evolutionary rate difference between *P. vulgaris *and *G. max *or that between *L. japonicus *and *M. truncatula*.

## Abbreviations

IR, inverted repeat; SSC, small single copy; LSC, large single copy; *ycf*, hypothetical chloroplast reading frame; *rrn*, ribosomal RNA; cpDNA, chloroplast genomic DNA; CDS, coding sequences; EST, expressed sequence tags; SNPs, single nucleotide polymorphisms; K, the number of nucleotide substitutions per site; Ka, the number of nucleotide substitutions per nonsynonymous site; Ks, the number of nucleotide substitutions per synonymous site; ω, the index of Ka/Ks; SDRs, short dispersed repeats; TRs, tandem repeats;

## Supplementary Material

Additional file 1 Average synonymous (Ks) and nonsynonymous (Ka) substitution rates ofprotein-coding genes in the *P. vulgaris *or *G. max *plastomes. The data show average synonymous (Ks) and nonsynonymous (Ka) substitution rates of 75 protein-coding genes derived from comparing *P. vulgaris *or *G. max *plastomes with the reference plastomes of *A. thaliana*, *L. japonicus *or *M. truncatula*. Click here for file

Additional file 2 Primers used for amplifying the complete plastome of the common bean. This file provides the sequences of primers used for amplifying the overlapped PCR products covering the complete plastome of the common bean. Click here for file
