# Supplementary material for: Rapid evolutionary change of common bean (Phaseolus vulgaris L) plastome, and the genomic diversification of legume chloroplasts
Source: BMC Genomics. 2007 Jul 10;8:228. doi: 10.1186/1471-2164-8-228 (PMC1940014; doi:10.1186/1471-2164-8-228)
Supplement: Additional file 1 — Average synonymous (Ks) and nonsynonymous (Ka) substitution rates ofprotein-coding genes in the P. vulgaris or G. max plastomes. The data show average synonymous (Ks) and nonsynonymous (Ka) substitution rates of 75 protein-coding genes derived from comparing P. vulgaris or G. max plastomes with the reference plastomes of A. thaliana, L. japonicus or M. truncatula. [file 1471-2164-8-228-S1.doc]

**Additional files**

***Additional file 1****. Average synonymous (Ks) and nonsynonymous (Ka) substitution rates of 75 protein-coding genes in P. vulgaris or G. max plastomes comparing with the reference plastomes*.

|  |  | *Arabidopsis* as reference | *Lotus* as reference | *Medicago* as reference |
| --- | --- | --- | --- | --- |
|  | *Phaseolus* | 0.0681 | 0.0446 | 0.0563 |
| Ka |  | (0.0392*) | (0.0984) | (0.2240) |
|  | *Glycine* | 0.0588 | 0.0348 | 0.0463 |
|  |  | (0.0389) | (0.0988) | (0.2096) |
|  | *Phaseolus* | 0.5657 | 0.3171 | 0.3048 |
| Ks |  | (0.0754) | (0.1059) | (0.1790) |
|  | *Glycine* | 0.5221 | 0.2762 | 0.2662 |
|  |  | (0.0953) | (0.1106) | (0.1602) |

* The data in parenthesis indicate the Standard deviations.
