# Supplementary material for: Rapid evolutionary change of common bean (Phaseolus vulgaris L) plastome, and the genomic diversification of legume chloroplasts
Source: BMC Genomics. 2007 Jul 10;8:228. doi: 10.1186/1471-2164-8-228 (PMC1940014; doi:10.1186/1471-2164-8-228)
Supplement: Additional file 2 — Primers used for amplifying the complete plastome of the common bean. This file provides the sequences of primers used for amplifying the overlapped PCR products covering the complete plastome of the common bean. [file 1471-2164-8-228-S2.doc]

**Additional files**

**Additional file 2** Primers used for amplifying the overlapped PCR products covering the complete plastome of common bean.

| **Names of pairs of oligos in each PCR reaction** | **Sequences of oligos (5’→3’)** | **Sizes of products (kb) of each PCR reaction** |
| --- | --- | --- |
| Clp1 | aatgcgagaatttgaggac |  |
| Clp2 | caaacaattcgattgggg | 7.5 |
|  |  |  |
| Clp3 | aaaggggattggttgaatag |  |
| Clp4 | gggtgggcaaggaatatc | 8.6 |
|  |  |  |
| Clp5 | cgataaccggcttttctc |  |
| Clp6 | ttttggcggagaaactatg | 11.4 |
|  |  |  |
| Clp7 | gaggttctaaccaaggag |  |
| Clp8 | ccagatcggctaattgttc | 12.1 |
|  |  |  |
| Clp9 | atcttggaaacacagcatac |  |
| Clp10 | ccctgttgattgagaatgg | 10.8 |
|  |  |  |
| Clp11 | aggaagaggccattgaac |  |
| Clp12 | ttcatcaactggcgctac | 8.3 |
|  |  |  |
| Clp13 | agagttattttcattctcaaatctg |  |
| Clp14 | tctcagtcgactcgctttt | 8.8 |
|  |  |  |
| Clp15 | gcgctacatcccttttcc |  |
| Clp16 | tccttggggttttcctg | 15 |
|  |  |  |
| Clp17 | tcctcggaagtttagagtag |  |
| Clp18 | ttcgggaaatttacaaacg | 12.8 |
|  |  |  |
| Clp19 | aactctgtgatagccatttc |  |
| Clp20 | actacttcactatcggtcac | 8.5 |
|  |  |  |
| Clp21 | agagctatacaagtgtcgtg |  |
| Clp22 | ccgtatttgggcttttcc | 7.1 |
|  |  |  |
| Clp23 | acaagaatccaaattcaatacg |  |
| Clp24 | aatggaatgagtttgagtatcg | 11.4 |
|  |  |  |
| Clp25 | atccctttgcattttgttc |  |
| Clp23 | acaagaatccaaattcaatacg | 8.2 |
|  |  |  |
| Clp16 | tccttggggttttcctg |  |
| Clp26 | agcttgtaaaaccacgactg | 8.5 |
